# Mobile App for Symptom Management and Associated Quality of Life During Systemic Treatment in Early Stage Breast Cancer: Nonrandomized Controlled Prospective Cohort Study

**DOI:** 10.2196/17408

**Published:** 2020-08-04

**Authors:** Cvetka Grašič Kuhar, Tjaša Gortnar Cepeda, Timotej Kovač, Matjaž Kukar, Nina Ružić Gorenjec

**Affiliations:** 1 Department of Medical Oncology Institute of Oncology Ljubljana Ljubljana Slovenia; 2 Faculty of Medicine Ljubljana University of Ljubljana Ljubljana Slovenia; 3 Faculty of Computer and Information Science University of Ljubljana Ljubljana Slovenia; 4 Institute for Biostatistics and Medical Informatics Faculty of Medicine Ljubljana University of Ljubljana Ljubljana Slovenia

**Keywords:** breast cancer, systemic therapy, mobile application, patient-reported outcome, quality of life

## Abstract

**Background:**

Providing patients with cancer who are undergoing systemic therapy with useful information about symptom management is essential to prevent unnecessary deterioration of quality of life.

**Objective:**

The aim was to evaluate whether use of an app for symptom management was associated with any change in patient quality of life or use of health resources.

**Methods:**

Outpatients with early stage breast cancer receiving systemic therapy were recruited at the Institute of Oncology in Ljubljana, Slovenia. Patients who received systemic therapy between December 2017 and March 2018 (control group) and between April 2018 and September 2018 (intervention group) were eligible. All patients received standard care, but only those in the intervention group were asked to use mPRO Mamma, an Android-based smartphone app, in addition. The app supported daily tracking of 50 symptoms, allowed users to grade their symptom severity (as mild, moderate, or severe), and also provided in-depth descriptions and recommendations based on reported symptom level. Patient-reported outcomes in both groups were assessed through the European Organisation for Research and Treatment of Cancer (EORTC) core (C-30) and breast cancer (BR-23) questionnaires, as well as a questionnaire about health resources use. The primary outcomes were the difference in the global quality of life between groups and the difference in summary score of the EORTC C-30 questionnaire between groups after 3 time periods (the first week of treatment, the first treatment cycle, and the entire treatment). The secondary outcome was the use of health resources (doctor visits and hospitalizations) in each time period. Other scales were used for exploratory analysis.

**Results:**

The mean difference between the intervention group (n=46) and the control group (n=45) in global quality of life (adjusted for baseline and type of surgery) after the first week was 10.1 (95% CI 1.8 to 18.5, *P*=.02). The intervention group summary scores were significantly higher than those of the control group after the first week (adjusted mean difference: 8.9, 95% CI 3.1 to 14.7, *P*=.003) and at the end of treatment (adjusted mean difference: 10.6, 95% CI 3.9 to 17.3, *P*=.002). Use of health resources was not statistically significant between the groups in either the first week (*P*=.12) or the first treatment cycle (*P*=.13). Exploratory analysis findings demonstrated clinically important improvements (indicated by EORTC C-30 or BR-23 scale scores)—social, physical, role, and cognitive function were improved while pain, appetite loss, and systemic therapy side effects were reduced.

**Conclusions:**

Use of the app enabled patients undergoing systemic therapy for early stage breast cancer to better cope with symptoms which was demonstrated by a better global quality of life and summary score after the first week and by a better summary score at the end of treatment in the intervention group compared to those of the control group, but no change in the use of health resources was demonstrated.

## Introduction

In the context of personalized treatment of a patient with cancer, the patient-reported outcome is gaining growing importance. This is because the physician’s ability to evaluate the patient’s subjective symptoms is not optimal. Physicians often underestimate the severity and frequency of symptoms experienced by patients during chemotherapy. Fromme et al [1] analyzed physician and patient reports on 8 symptoms in a metastatic prostate cancer chemotherapy trial and found that the physician reports had low sensitivity and specificity for detecting chemotherapy side effects [1]. Moreover, the agreement between multiple physicians was, at best, moderate [2]. Physician assessment of symptom presence and severity differed by one to two grades according to the Common Toxicity Criteria of Adverse Effects [3] which, the study [2] concluded, would have resulted in different treatment decisions (such as a difference between continuing chemotherapy, halting chemotherapy, or changing treatment dosage). To better assess patient safety and toxicity in clinical studies of treatments, the National Cancer Institute of the United States of America developed a patient-reported outcome version of the Common Toxicity Criteria of Adverse Effects (PRO-CTCAE), which consists of 78 adverse effects criteria that are appropriate for patient self-reporting. For each adverse effect, one or more descriptive measures such as the adverse effect severity, frequency, and interference with daily activity are assessed yielding 124 PRO-CTCAE items, each graded on a 5-point scale (from 0 to 4, where 0 indicates the adverse effect is absent, and 4 indicates the most disabling adverse effect) [4]. An appropriate subset of these items should be selected for each clinical trial; however, most patients are treated outside of clinical trials.

With the widespread use of smartphones, electronic collection of patient-reported outcomes is becoming possible. Several studies [5-12] have shown the feasibility and clinical utility of electronic capture and monitoring of patient-reported outcome for symptoms, functional status, and quality of life during systemic treatment of cancer. In advanced solid tumors, Basch et al [5] showed improved health-related quality of life measures and reduced emergency room admittance in patients who received weekly notifications and reported their symptoms (also weekly) [5]. Integration of the patient-reported outcome into routine care was even shown to prolong overall survival in patients with advanced solid tumors by 5 months [13]. With increased patient willingness to use technology, there are possibilities for expansion of patient-centered medicine [14-16]. At present, few apps are used for the self-management of symptoms [7,9-11,14]. In one study [10], higher cognitive function and a high symptom burden in patients were predictive factors for whether they would use symptom self-management strategies.

The aim of our study was to assess whether the use of our innovative mobile app by patients with early stage breast cancer who were receiving systemic treatment was associated with any changes in their health-related quality of life (global quality of life, ability to function in daily life, symptom severity) and in the use of health resources. Our hypothesis was that the mobile app users would have better health-related quality of life outcomes, especially after the first week and after the first cycle of chemotherapy, and would use fewer health resources.

## Methods

### Study Design and Population

We conducted a prospective nonrandomized controlled study with a cohort consisting of outpatients who were being treated with systemic therapy (chemotherapy) for early stage breast cancer at the Institute of Oncology in Ljubljana. The study protocol was approved by the Ethical Commission at the Institute of Oncology and Commission of the Republic of Slovenia for Medical Ethics (0120-386/2017/3). The researchers followed the principles of the Declaration of Helsinki.

Patients were recruited after their first appointment with a medical oncologist. All study information was provided by oncologists, medical residents, and medical students working on the project. Patients were eligible if they were scheduled to receive neoadjuvant or adjuvant chemotherapy (and anti–human epidermal growth factor 2 targeted therapy, if indicated) for early stage breast cancer, their smartphone was Android-based, and they were willing to fill in paper and pencil questionnaires reporting their quality of life while receiving treatment. The patients were ineligible if their smartphone was Windows-based or an iPhone, they were unfamiliar with using a smartphone, or they were unable to communicate effectively in Slovenian. All participants provided informed consent prior to being enrolled in the study.

### Study Group and Intervention

The control group consisted of patients enrolled between December 2017 and March 2018; the intervention group consisted of patients enrolled between April 2018 and September 2018 (since the mobile app was not finished until April). The control group received the standard information about chemotherapy side effects. The intervention group received both the standard information and access to the mPRO Mamma mobile app [17] which was accompanied by a detailed user instruction guide.

### Study Procedures

Patients in both groups were asked to complete the European Organisation for Research and Treatment of Cancer Quality of Life Questionnaire Core 30 (EORTC QLQ C-30) and Quality of Life Questionnaire Breast Cancer Module (EORTC QLQ BR-23) [18,19] and a questionnaire about health resources use at 4 time points: before systemic therapy (baseline), after the first week, after the first cycle, and at the end of systemic therapy. Upon enrollment, each patient received questionnaires (4 sets) along with instructions to return completed questionnaires by mail in a timely manner. The patients who returned the baseline questionnaires and questionnaires from at least one other time point were included in the analysis.

### Mobile App

**Figure 1:**
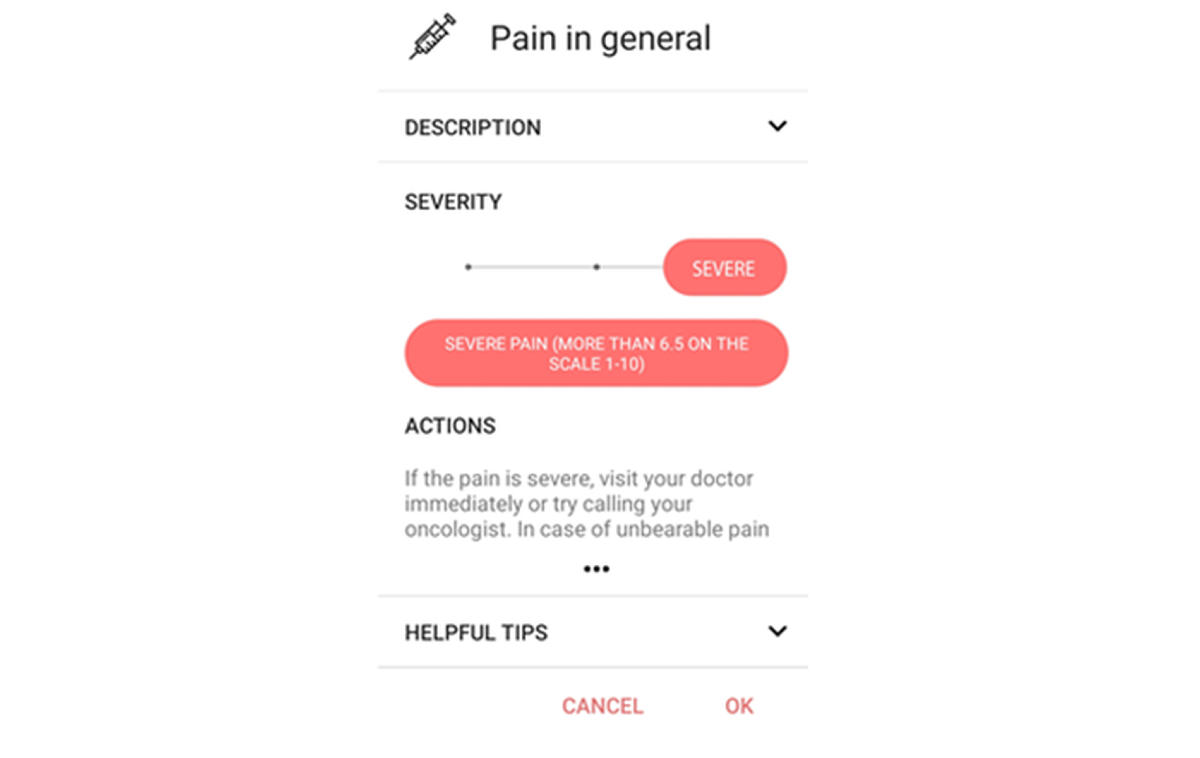
The mobile app user interface with customized slider controls allows for quick daily recording of symptoms and their severity levels.

For the purpose of this study, we developed an Android mobile app called mPRO Mamma [17]. It conforms to standard Google Material Design guidelines [20], but additionally features customized slider controls to visually indicate symptom severity levels (Figure 1). It allows for quick daily recording of symptoms and symptom severity (with reminder notifications) and can send encrypted reports to the patient’s oncologist, if selected. The app was designed and revised several times in cooperation with experienced medical oncologists, medical students, and computer scientists. It has been implemented according to the best principles of Material Design multilingual support and currently supports Slovenian and English.

We selected 50 symptoms that were relevant to the expected adverse effects of either cytotoxic regimens or targeted drugs administered to patients during the neoadjuvant or adjuvant breast cancer treatments from the 78 symptoms of PRO-CTCAE. Each symptom had a description, a severity scale, and advice for its management. Patients labeled and reported a symptom only if it occurred. To make our app clear and useful, symptom severity was graded on a 3-point scale as mild, moderate, or severe.

For each grade of symptom severity, a health professional’s advice was presented. For mild or moderate ratings by the patient, suggestions that would allow them to alleviate the symptoms on their own were displayed. If symptoms were rated as severe, the app advised the patient to visit either their general practitioner or an emergency department. Patients reported their symptoms in the app on a daily basis and sent the symptom report to their treating oncologist as an encrypted email on the day before their next therapy cycle (every 2 or 3 weeks, depending on their chemotherapy schedule). Screenshots of the symptom entry page and daily report are presented in Multimedia Appendix 1, Multimedia Appendix 2, and Multimedia Appendix 3.

### Outcome Measures

The EORTC QLQ C-30 and BR-23 consist of a global quality of life score, and several functional and symptom scales, which can be combined in a summary score. Our primary outcomes were the global quality of life score and summary score at three time points (after the first week, after the first cycle, and at the end of treatment). Other scales were used in our exploratory analysis. The EORTC QLQ scoring manual was followed [21]. All scales had values from 0 to 100, where 100 represented the best global quality of life, the best functioning, or the worst symptoms. The summary score, which ranged from 0 (worst) to 100 (best), was calculated from 13 out of 15 EORTC QLQ-C30 scales (the global quality of life and the financial impact scale were excluded) in accordance with Giesinger et al [22] and instructions from the EORTC [21-23]. We used previously established thresholds [24] for important differences in quality of life—clinically meaningful difference was set to 10 points.

The secondary outcome was the use of health resources which was measured by self-reported number of visits to the doctor and number of hospitalizations in the first week and in the first cycle of therapy (questionnaires at the end of treatment were not included because they may not have been reliable due to the long time span between the start and the end of treatment).

### Statistical Analysis

#### Sample Size Determination

A sample size sufficient to detect a difference between the intervention and control groups for changes of the global quality of life scores from baseline scores using an independent *t* test was determined using a power analysis. For a significance level of α=.05 and 80% power to reject the null hypothesis of equal means of groups when the population mean difference is equal to 13 yielded a sample size of 90 (45 per group); a standard deviation of 22 was used for both groups based on the results from a recent randomized trial [25]. It was subsequently determined that a more advanced analysis had to be conducted, as explained below.

#### Data Analysis

Categorical variables (tumor size, axillary nodes, histologic type, tumor grade, estrogen receptors, progesterone receptors, HER2, Ki-67 expression, tumor subtype, type of surgery, breast reconstruction, chemotherapy, type of chemotherapy, doctor visits during first week, doctor visits during the first cycle, hospitalization during the first week, and hospitalization during the first cycle) were summarized with frequencies and percentages. Numerical variables (age, number of chemotherapy cycles, global quality of life score, and summary score) were described with means and standard deviations (or medians and interquartile ranges if distributions were asymmetric). To compare the intervention and control groups at baseline, chi-square tests (or Fisher exact test, if more than 20% of expected frequencies were below 5) were used for categorical variables, and two-tailed independent *t* tests (or Mann-Whitney *U* tests, if distributions were asymmetric) were used for numerical variables.

It was required that the model account for differences between the groups in the type of surgery (a higher proportion of mastectomies, and consequently, reconstruction in the control group), as well as in quality of life and summary score at baseline. The primary outcomes of global quality of life score and summary score were analyzed using linear mixed-effects models [26]. Linear mixed-effects models allowed for the use of one model to represent all three time points, adjusting for covariates at baseline and where a random intercept (indexed by patient ID) accounted for repeat measurements of the same patient. In each model, the fixed effects were time (after the first week, after the first cycle, or at the end of treatment), group (intervention or control), interaction between group and time, value at baseline, and type of surgery (breast-conserving surgery or mastectomy).

In the exploratory analysis, we examined all 19 other scales of the QLQ-C30 and the QLQ-BR23 (excluding sexual enjoyment and upset by hair loss, which were both dependent upon answering affirmative to other questions, and excluding financial difficulties). The change from baseline between the groups at each of the three time points was analyzed separately using the Mann-Whitney *U* test because of either distribution asymmetry or the low number of possible values of the scale. All *P* values in the exploratory analysis served only as an aid to evaluate the differences between the groups. Nevertheless, with so many hypotheses tested, the *P* values were adjusted using Holm method to control the family-wise error rate.

An (adjusted) *P*<.05 was considered as statistically significant. All analyses were performed using R statistical software (version 3.4.3) [27].

## Results

### Patient Characteristics

**Figure 2:**
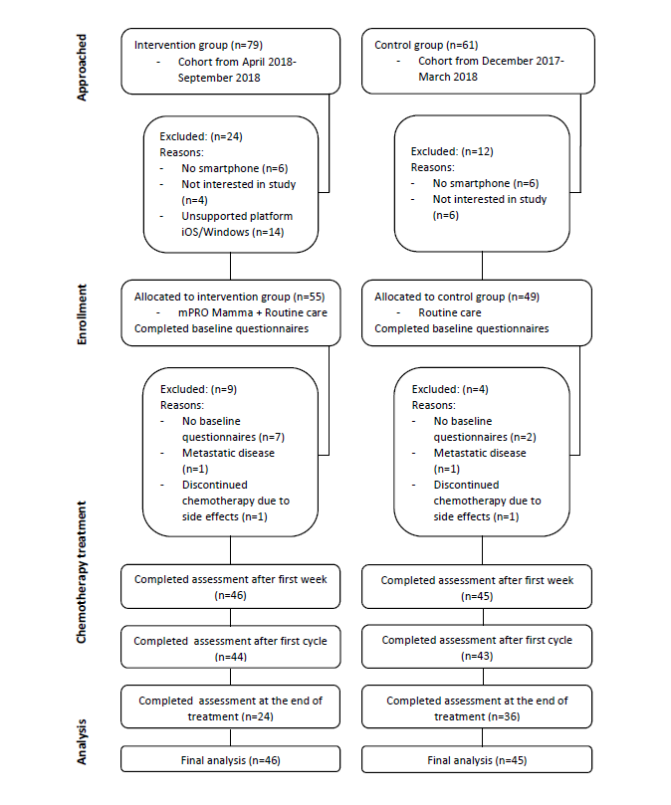
Consolidated Standard of Reporting Trials (CONSORT) diagram showing the flowchart of patients with breast cancer by group and reasons for exclusion from the study and the analysis.

The study flow chart is presented in Figure 2. We performed a CONSORT-EHEALTH checklist (Multimedia Appendix 4) according to pilot testing [28].

The demographic, clinical, and treatment data for the control (n=45) and the intervention (n=46) groups are presented in Table 1. Patients in both groups were similar with respect to age (t_89_=0.86, *P*=.39), tumor size (Fisher exact test, *P*=.48), nodal involvement (Fisher exact test, *P*=.13), histology (Fisher exact test, *P*=.42), tumor grade (Fisher exact test, *P*=.38), hormone receptors (progesterone: χ^2^_1_=1.23, *P*=.27; estrogen: χ^2^_1_=2.33, *P*=.13), human epidermal growth factor 2 status (χ^2^_1_=1.76, *P*=.19), tumor subtype (Fisher exact test, *P*=.30), type of chemotherapy (Fisher exact test, *P*=.41), and number of chemotherapy cycles (Mann-Whitney *U* test, *P*=.73); however, there was a significant difference between the intervention and control groups with respect to type of surgery (χ^2^_3_=12.60, *P*=.006). Patients in the control group had undergone more mastectomies, and consequently, had also undergone reconstruction more often (Fisher exact test, *P*=.01). At baseline, patients in the control group had lower quality of life and summary scores than those of the intervention group (quality of life: Mann-Whitney *U* test, *P*=.06; summary: Mann-Whitney *U* test, *P*=.02). Baseline differences in quality of life and summary scores and for type of surgery (mastectomy or not) between the groups were, therefore, taken into account and adjusted for, within the model.

**Table 1:** Patient characteristics in the intervention and control group.

Characteristics			Groups			Comparison between groups	
		All patients (n=91)	Intervention (n=46)	Control (n=45)	Chi-square^a^ or *t* test^b^ (*df*)		*P* value
Age in years, mean (SD)		51.7 (9.5)	50.9 (9.3)	52.6 (9.6)	0.86^b^ (89)		.39
Number of chemotherapy cycles, median (IQR)		6 (4-7)	6 (5-7)	6 (4-7)	N/A^c^		.73
Quality of life score at baseline, median (IQR)		66.7 (58.3-83.3)	83.3 (66.7-91.7)	66.7 (58.3-83.3)	N/A^c^		.06
Summary score at baseline, median (IQR)		90.9 (83.8-96.2)	93.6 (86.2-97.9)	89.1 (83.3-93.7)	N/A^c^		.02
**Tumor size, n (%)**					N/A^d^		.48
	≤20 mm	43 (47)	24 (52)	19 (42)			
	21-50 mm	39 (43)	19 (41)	20 (44)			
	>50 mm	9 (10)	3 (7)	6 (13)			
**Axillary nodes, n (%)**					N/A^d^		.13
	Negative nodes	41 (45)	23 (50)	18 (40)			
	1-3 positive nodes	37 (41)	15 (33)	22 (49)			
	4-6 positive nodes	9 (10)	7 (15)	2 (4)			
	>6 positive nodes	4 (4)	1 (2)	3 (7)			
**Histologic type, n (%)**					N/A^d^		.42
	Invasive ductal carcinoma	77 (85)	40 (87)	37 (82)			
	Invasive lobular carcinoma	5 (6)	1 (2)	4 (9)			
	Other	9 (10)	5 (11)	4 (9)			
**Tumor grade, n (%)**					N/A^d^		.38^e^
	Grade I	2 (2)	0 (0)	2 (4)			
	Grade II	30 (33)	14 (30)	16 (36)			
	Grade III	58 (64)	31 (67)	27 (60)			
	Unknown	1 (1)	1 (2)	0 (0)			
**Estrogen receptors, n (%)**					2.33^a^ (1)		.13
	Negative	18 (20)	12 (26)	6 (13)			
	Positive	73 (80)	34 (74)	39 (87)			
**Progesterone receptors, n (%)**					1.23^a^ (1)		.27
	Negative	25 (28)	15 (33)	10 (22)			
	Positive	66 (73)	31 (67)	35 (78)			
**HER2^f^ , n (%)**					1.76^a^ (1)		.19
	Negative	65 (71)	30 (65)	35 (78)			
	Positive	26 (29)	16 (35)	10 (22)			
**Ki-67^g^ expression, n (%)**					1.69^a^ (1)		.19
	Negative (≤15 %)	21 (23)	8 (17)	13 (29)			
	Positive (> 15 %)	70 (77)	38 (83)	32 (71)			
**Tumor subtype, n (%)**					N/A^d^		.30
	Luminal A–like	9 (10)	4 (9)	5 (11)			
	Luminal B HER2 negative	43 (47)	20 (44)	23 (51)			
	Luminal B HER2 positive	17 (19)	8 (17)	9 (20)			
	HER2 positive	8 (9)	7 (15)	1 (2)			
	Triple negative	14 (15)	7 (15)	7 (16)			
**Type of surgery, n (%)**					12.60^a^ (3)		.006
	Breast conserving surgery + SNB^h^	36 (40)	26 (57)	10 (22)			
	Breast conserving surgery + axillary lymphadenectomy	12 (13)	6 (13)	6 (13)			
	Mastectomy + SNB	21 (23)	6 (13)	15 (33)			
	Mastectomy + axillary dissection	22 (24)	8 (17)	14 (31)			
**Breast reconstruction, n (%)**					N/A^d^		.01
	No	70 (77)	41 (89)	29 (64)			
	Deep inferior flap	12 (13)	2 (4)	10 (22)			
	Tissue expander	9 (10)	3 (7)	6 (13)			
**Chemotherapy, n (%)**					0.38^a^ (1)		.54
	Adjuvant chemotherapy	64 (69)	31 (67)	33 (73)			
	Neoadjuvant chemotherapy	27 (30)	15 (33)	12 (27)			
**Type of chemotherapy, n (%)**					N/A^d^		.41
	Anthracycline + taxanes	63 (69)	35 (76)	28 (62)			
	Anthracyclines alone	17 (19)	6 (13)	11(24)			
	Taxanes alone	8 (9)	3 (7)	5 (11)			
	CMF^i^	3 (3)	2 (4)	1 (2)			

^a^Denotes a chi-square test value.

^b^Denotes a *t* test value.

^c^N/A: not applicable; in this instance, denotes the use of Mann-Whitney *U* test.

^d^N/A: not applicable; in this instance; denotes the use of Fisher exact test.

^e^Unknown values were excluded from calculations.

^f^HER2: human epidermal growth factor 2.

^g^Ki-67: proliferation marker.

^h^SNB: sentinel lymph node biopsy.

^i^CMF: cyclophosphamide, methotrexate, fluorouracil.

### Primary Outcome Measures

In Table 2 and in Table 3, we have presented only effects that are of interest (ie, adjusted mean difference between the groups for each of the 3 time periods with 95% confidence intervals and *P* values; for all mean differences, a positive difference indicates a higher value in the intervention group than the corresponding value in the control group). The adjusted mean differences between the intervention and the control group after the first week of chemotherapy were statistically significant for both the global quality of life score (10.1, 95% CI 1.8 to 18.5, *P*=.02) and the summary score (8.9, 95% CI 3.1 to 14.7, *P*=.003). The adjusted mean difference for summary score at the end of treatment (10.6, 95% CI 3.9 to 17.3, *P*=.002) was also significant. Complete model results are presented in Multimedia Appendix 5 and Multimedia Appendix 6. Clinically meaningful differences in the adjusted means between groups were found for global quality of life score after the first week and for summary score at the end of treatment (ie, an adjusted mean difference greater than 10).

**Table 2:** Global quality of life score adjusted mean differences between intervention and control groups.

Time period	Estimate	95% CI	*P* value
First week	10.1	1.8 to 18.5	.02
First treatment cycle	4.7	–3.8 to 13.2	.27
Entire treatment period	7.0	–2.7 to 16.7	.16

**Table 3:** Summary score adjusted mean differences between intervention and control groups.

Time period	Estimate	95% CI	*P* value
First week	8.9	3.1 to 14.7	.003
First treatment cycle	5.3	–0.6 to 11.2	.08
Entire treatment period	10.6	3.9 to 17.3	.002

### Secondary Outcome Measures

In the first week, 44% (20/46) of patients in the intervention group visited the doctor, and 60% (27/45) of patients in the control group visited the doctor. In the first cycle, 37% (16/43) of patients in the intervention group visited the doctor at least twice, and 54% (21/39) of patients in the control group visited the doctor at least twice. The differences between groups were not statistically significant (first week: χ^2^_1_=2.49, *P*=.12; first cycle: χ^2^_1_=2.29, *P*=.13; Table 4). The number of hospitalizations was low—3% (3/91) in the first week and 7% (6/84) in the first cycle—without substantial or statistically significant differences between the groups (first week: Fisher exact test, *P*=.62; first cycle: Fisher exact test, *P*>.999).

**Table 4:** Health care use during the first week and the first cycle of systemic therapy.

		All, n (%)	Intervention group, n (%)	Control group, n (%)	Chi-square^a^ (*df*)	*P* value
**Doctor visits during first week**		91 (100)	46 (100)	45 (100)	2.49^a^ (1)	.12
	no visits	44 (48)	26 (56)	18 (40)		
	1 visit or more	47 (52)	20 (44)	27 (60)		
**Doctor visits during first cycle**		82 (100)	43 (100)	39 (100)	2.29^a^ (1)	.13
	1 visit or less	45 (55)	27 (63)	18 (46)		
	2 or more visits	37 (45)	16 (37)	21 (54)		
**Hospitalizations during first week**		91 (100)	46 (100)	45 (100)	N/A^b^	.62
	No	88 (97)	45 (98)	43 (96)		
	Yes	3 (3)	1 (2)	2 (4)		
**Hospitalizations during first cycle**		84 (100)	43 (100)	41 (100)	N/A^b^	>.999
	No	78 (93)	40 (93)	38 (93)		
	Yes	6 (7)	3 (7)	3 (7)		

^a^Denotes a chi-square test value.

^b^N/A: not applicable; in this instance, denotes the use of Fisher exact test.

### Exploratory Analysis of Other EORTC QLQ C-30 and BR-23 Scales

**Figure 3:**
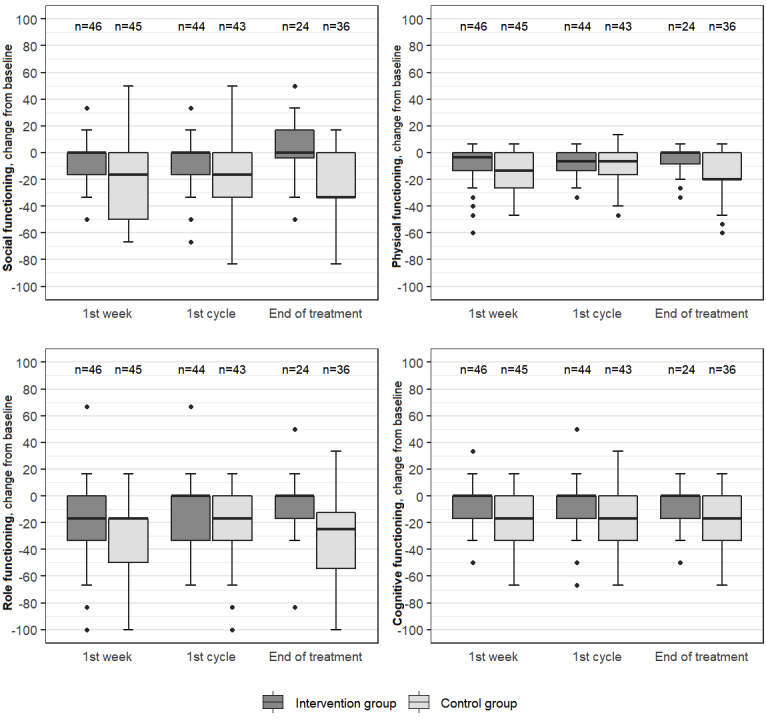
Boxplots showing change from baseline for the selected functional scales of the EORTC QLQ C-30 and BR-23. EORTC: European Organisation for Research and Treatment of Cancer; QLQ BR-23: Quality of Life Questionnaire Breast Cancer Module; QLQ C-30: Quality of Life Questionnaire Core 30.

**Figure 4:**
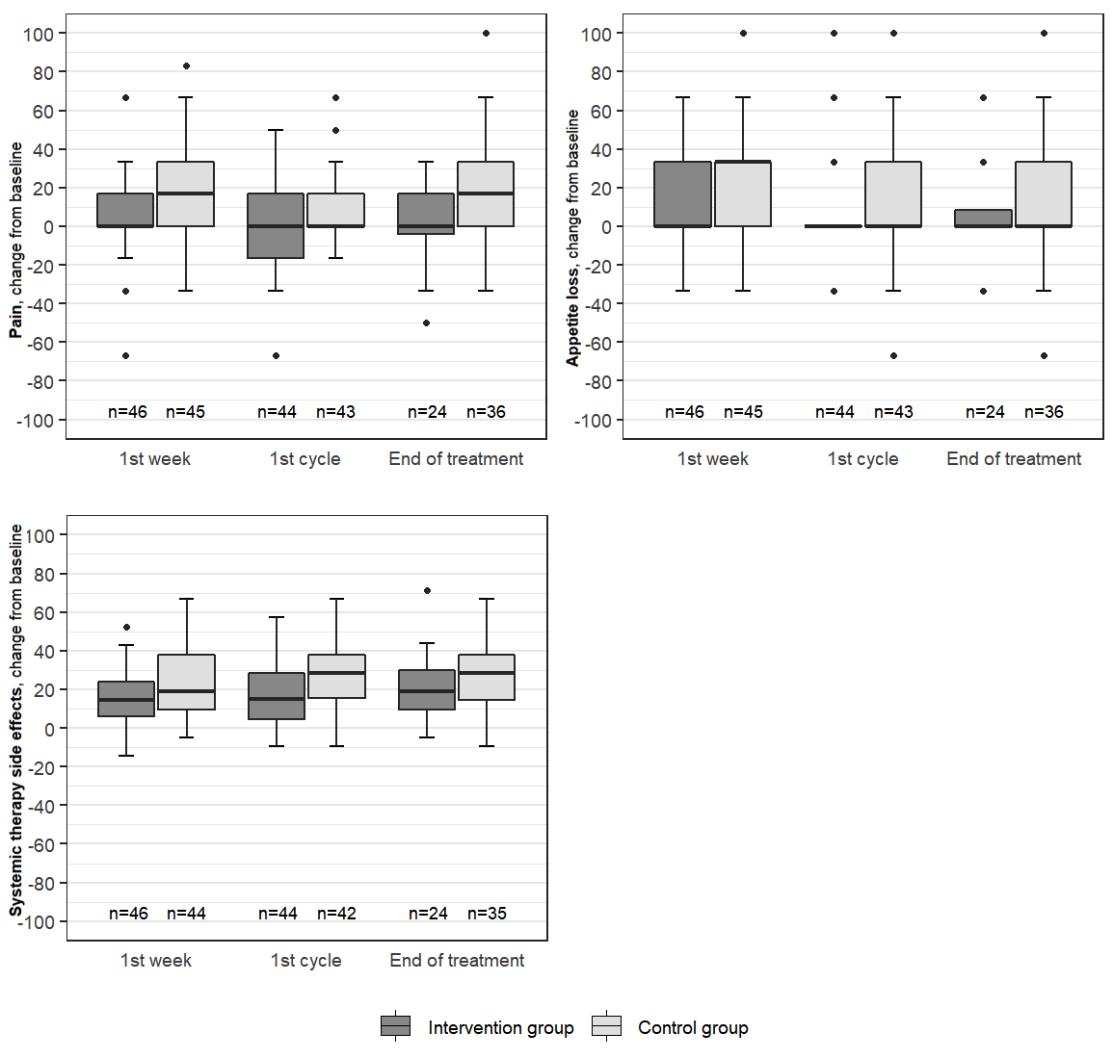
Boxplots showing change from baseline for the selected symptom scales of EORTC QLQ C-30 and BR-23. EORTC: European Organisation for Research and Treatment of Cancer; QLQ BR-23: Quality of Life Questionnaire Breast Cancer Module; QLQ C-30: Quality of Life Questionnaire Core 30.

Scales with clinically important differences between the groups (baseline adjusted), and with *P*<.10 (Mann-Whitney *U* tests) were social functioning (first week: *P*=.001, adjusted *P*=.04; first cycle: *P*=.005, adjusted *P*=.29; end of treatment: *P*=.003, adjusted *P*=.14), pain (first week: *P*=.03, adjusted *P*>.999; end of treatment: *P*=.005, adjusted *P*=.29), physical functioning (first week: *P*=.051, adjusted *P*>.999; end of treatment: *P*=.005, adjusted *P*=.27), role functioning (end of treatment: *P*=.01, adjusted *P*=.49), cognitive functioning (end of treatment: *P*=.09, adjusted *P*>.999), appetite loss (first week: *P*=.04, adjusted *P*>.999), and systemic therapy side effects (first cycle: *P*=.08, adjusted *P*>.999). These scales are presented in Figures 3 and 4. Complete exploratory analysis results are available in the Multimedia Appendix 7. The only difference between the groups that could be generalized to the population after adjusting for multiple testing was in social functioning after the first week (adjusted *P*=.04).

## Discussion

### Principal Findings

In this study, we investigated the impact of using of a mobile app for symptom reporting and self-management on patient-reported outcomes in early stage breast cancer patients who were receiving chemotherapy. Chemotherapy can cause numerous adverse effects which have a negative impact on health-related quality of life. Our hypothesis was that the intervention group (those using the mobile app) would cope better with adverse effects, which would result in the improved global quality of life and summary scores, especially at the beginning of chemotherapy, and would result in reduced use of health resources.

Our first hypothesis about the improved global quality of life and summary score in the intervention group using the mobile app was confirmed. After the first week of treatment, global quality of life reported by the intervention group was better than that reported by the control group, with clinically important mean differences (Table 2). The global quality of life score represented patient perception of overall health and overall quality of life in the previous week, but was not sensitive enough to detect group differences over time [22]. This was confirmed in our analysis as both groups had similar global quality of life scores after the first cycle and at the end of treatment. Summary score, however, was a more robust alternative to the global quality of life score, as it took into account 13 scales that were calculated from 27 out of 30 EORTC QLQ C-30 items. We found statistically significant differences between groups in summary scores after the first week and at the end of treatment; the latter demonstrated a clinically important difference (Table 3). Our mobile app assisted patients as an interventional tool for the self-management of symptoms. As the patient reported a symptom, the mobile app automatically provided the most suitable information for self-management according to the reported level of severity (Multimedia Appendix 1 and Multimedia Appendix 2). This improved patient operative capability and patient self-efficacy, which resulted in an improved quality of life in accordance with Bandura’s theory of self-efficacy theory [29].

To investigate which EORTC QLQ C-30 scales most significantly contributed to the difference in summary score, we performed an exploratory analysis. QLQ BR-23 scales were also included in this analysis to shed light on symptoms and functioning that were specific to breast cancer. We found clinically important improvements in social, physical, role, and cognitive functioning in the intervention group versus the control group, as well as less severe pain, less appetite loss, and fewer systemic therapy side effects in the intervention group (Multimedia Appendix 7, Figure 3, and Figure 4). In a recently published paper on lung cancer [30], patients with access to a web-based health education program reported better global quality of life and emotional functioning, as well as a significant decrease in ten of the most important symptoms compared to those in the control group; however, no differences in physical, role, cognitive, or social functioning were found [30]. Patients with lung cancer, however, cannot be directly compared to patients with breast cancer. To our knowledge, comparable studies for breast cancer patients do not exist. We believe that the intervention group had better social, physical, role, and cognitive functioning due to the use of the mobile app (better self-management). For cancer patients, solving the problem (ie, self-managing of symptoms) correlates significantly with improvements in their levels of psychological distress, overall quality of life, role, and physical functioning [31,32].

We found the strongest effect was for social functioning after the first week, as the difference between groups remained statistically significant even after adjusting for multiple comparisons. Patients who receive chemotherapy treatments experience imposed limitations in social functioning, which is also influenced by family support, outlook on life and opportunities for social exchange [33]. The mobile app had a positive impact on social functioning, although, a bias as a result of nonrandom patient allocation may have been present.

Less severe systemic therapy side effects, pain, and appetite loss in the intervention group were probably due to self-management techniques employed by these patients. On the other hand, the surgery-related group differences may have contributed.

Our second hypothesis was not confirmed. We did not find any statistically significant difference between groups for use of health resources. Self-management strategies employed by patients themselves probably resolved mild and moderate side effects. For severe symptoms, we presumed that an option of immediate triggering alert to medical staff at its onset would reduce health resource use. Basch et al [5] reported significantly lower health resource usage in the group allocated to symptom reporting, probably because a severe symptom grade triggered an email alert to nurses.

Ginossar et al [34] performed a systematic review of literature and found over 100 breast cancer-related mobile apps; however, many of them did not lead to behavior change and many were not evidence-based. Recent mobile apps for women with breast cancer have a positive effect by promoting weight loss, decreasing stress, and improving the quality of life [35,36]. Apps for reporting symptoms and promoting self-care during cancer chemotherapy treatment are still rare [6-11,37,38]. Electronic capture and monitoring of patient-reported outcome for symptoms during systemic treatment of cancer has been found to be feasible, even though patients were not provided with feedback about their management [5]; however, in studies [6,7,9,10] where automated alerts for self-management of reported symptoms were available, a rapid benefit in the form of decreased symptom severity has been identified. It has been reported [6] that automated alerts were more effective than nurse-administrated symptom management. In our study, we also rapidly found a benefit from the use of our mobile app, which was demonstrated by better global quality of life and summary scores in the first week after receiving chemotherapy. In our opinion, the first week after chemotherapy is a very important time to alleviate distress in patients. A recently published randomized trial from Japan [37] tested a mobile app similar to ours, to see whether the app affected anxiety and depression, but no improvement in anxiety, depression, or health literacy were found at the end of treatment [37].

Using a mobile app in collaboration with the treating physician improved patient well-being and their awareness of chemotherapy adverse effects [38]; however, the impact of the mobile app on quality of life outcomes was not assessed [37,38]. Zhu et al [9] reported findings similar to ours, namely that the e-support group had better quality of life, self-efficacy, and symptom control than that of the group receiving standard care. They found beneficial effects at 3 months, but which disappeared at 6 months [9]. In our study, even at the end of treatment (approximately 6 months), the beneficial effect in summary score (but not in global quality of life) remained; however, Zhu et al [9] assessed quality of life using a different questionnaire, ie, the Functional Assessment of Cancer Treatment-B. Recently, a mobile phone–based system for the remote monitoring and management of chemotherapy-related side effects was evaluated in Canadian patients with cancer; however, its impact on health-related quality of life has not yet been tested [11]. Several randomized controlled trials evaluating remote electronic monitoring and symptom management are still ongoing [14-16].

### Strengths

The advantage of our study was the detailed patient-reported outcome analysis of the effect of mobile app usage, with data collected using validated questionnaires (EORTC QLQ C-30 and BR-23). In addition to using a standard tool (global quality of life score), we used summary score, a new tool suggested by EORTC. To our knowledge, our study is the first that uses both quality of life and summary score as primary outcomes when analyzing the impact of a mobile app on the care of patients with breast cancer who are receiving chemotherapy. Moreover, we appropriately planned the first assessment for after the first week, indicated by the statistically significant difference in the global quality of life and summary score at this time.

### Limitations

Our study has some limitations. First, it was a nonrandomized controlled cohort study. In order to avoid potential bias, it would have been better to conduct a randomized controlled trial. In fact, the groups in our study differed with respect to type of breast surgery, quality of life score, and summary score at baseline, so the results were adjusted for these differences using appropriate statistical methods. There may also have been seasonal differences affecting patient well-being since patients were enrolled at different times of the year. In addition, a possible bias may have existed as a result of different recruitment techniques used by the medical oncologists. At the beginning of the clinical study, we were still in the process of programming our mobile app which prevented us from randomly allocating patients.

Second, not all symptoms that can arise during the systemic therapy can be assessed with the QLQ C-30 and BR-23 questionnaires. Our app included approximately 50 of the 78 recommended PRO-CTCAE symptoms, so instructions for patient self-management for the missing symptoms were not included in the app; however, we aimed to include the symptoms that occur most commonly over the course of breast cancer treatments. Our collection was broader than that used by Zhang et al [39] who developed and evaluated a patient-reported outcome scale for breast cancer containing 38 items [39].

Our app could also be improved by utilizing wearable sensors (smart bracelets, watches, rings, etc) and incorporating additional questionnaires capable of measuring psychological distress, mental distress, and anxiety; it could be also equipped with alerts reminding patients to provide data at predetermined dates.

### Future Work

Based on the positive effects on health-related quality of life measured in the systemic therapy part of breast cancer treatment, we plan to include other treatment modalities for breast cancer patients, ie, surgical interventions and radiotherapy. Another interesting upgrade would be to include a follow-up period. Our aim is to provide regular updates to ensure software compatibility as well as to avoid potential security issues. Since our app is likely most useful for mild and moderate symptoms where patients are empowered to manage symptoms themselves, we plan to also make the app useful for patients with severe symptoms. We could establish a system where alerts about patient symptoms are sent to a dedicated hospital server. If the alert contained any severe symptoms, it would be labelled as urgent and an appointed research nurse would be trained to react in accordance with the prespecified medical algorithms. Such algorithms are also incorporated in recently reported proposals of studies [14-16] to assess symptom burden, quality of life, supportive care needs, anxiety, self-care, self-efficacy, work limitations, and cost effectiveness.

Our findings regarding the impact of mobile app use on the quality of life and summary scores in combination with those regarding other scales that were only a part of our exploratory analysis, should be confirmed in a larger randomized controlled trial. Further research is also needed in order to extend these findings to a broader population of patients, including those suffering from other ailments, and to different organizations in the medical field.

### Conclusions

Our mobile app has interventional value for the self-management of symptoms for patients with breast cancer who are receiving systemic therapy which was shown by a better global quality of life scores (in the first week of therapy) and a better summary scores (in the first week and at end of treatment) for the intervention group than for the control group. Based on the exploratory analysis, the app contributed to clinically important improvements in social, physical, role, and cognitive functioning while diminishing pain, appetite loss, and systemic therapy side effects.

## Appendix

Multimedia Appendix 1 Screenshot of a page for the daily report of a symptom pain.

Multimedia Appendix 2 Screenshot of a page for the daily report of symptoms of nausea and vomiting.

Multimedia Appendix 3 Screenshot of a page for the daily report of symptoms with their severity.

Multimedia Appendix 4 CONSORT-eHEALTH checklist (V 1.6.1) in pilot-testing.

Multimedia Appendix 5 Linear mixed-effects model for global quality of life, whole model.

Multimedia Appendix 6 Linear mixed-effects model for summary score, whole model.

Multimedia Appendix 7 The explorative analysis of the other scales of the EORTC QLQ C-30 and BR-23.

## Abbreviations

EORTC: European Organisation for Research and Treatment of Cancer
HER2: human epidermal growth factor 2
Ki-67: proliferation marker
PRO-CTCAE: patient-reported outcome version of the Common Toxicity Criteria of Adverse Effects
QLQ BR-23: Quality of Life Questionnaire Breast Cancer Module
QLQ C-30: Quality of Life Questionnaire Core 30
