# Supplementary figures and images for: Mobile App for Symptom Management and Associated Quality of Life During Systemic Treatment in Early Stage Breast Cancer: Nonrandomized Controlled Prospective Cohort Study
Source: JMIR Mhealth Uhealth. 2020 Aug 4;8(8):e17408. doi: 10.2196/17408 (PMC7435681; doi:10.2196/17408)

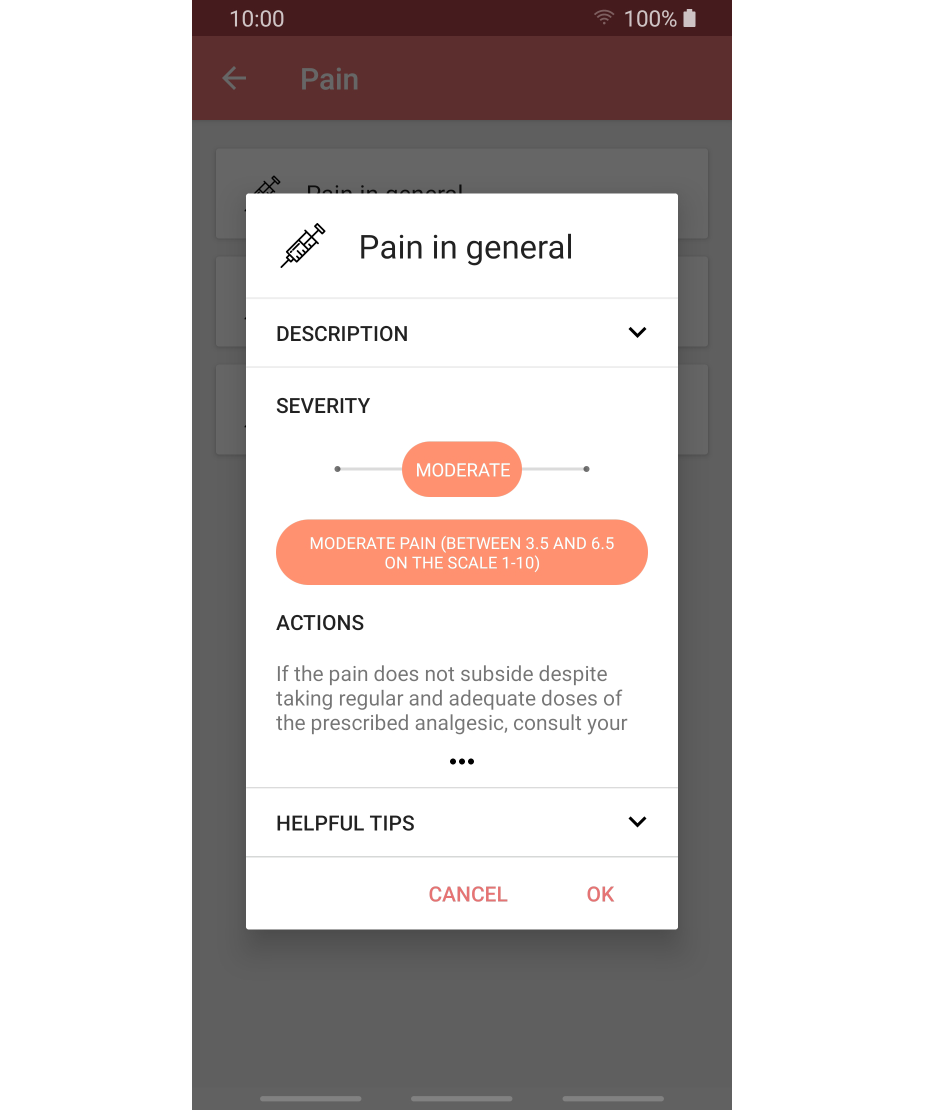

Supplement: Multimedia Appendix 1 [file mhealth_v8i8e17408_app1.png]

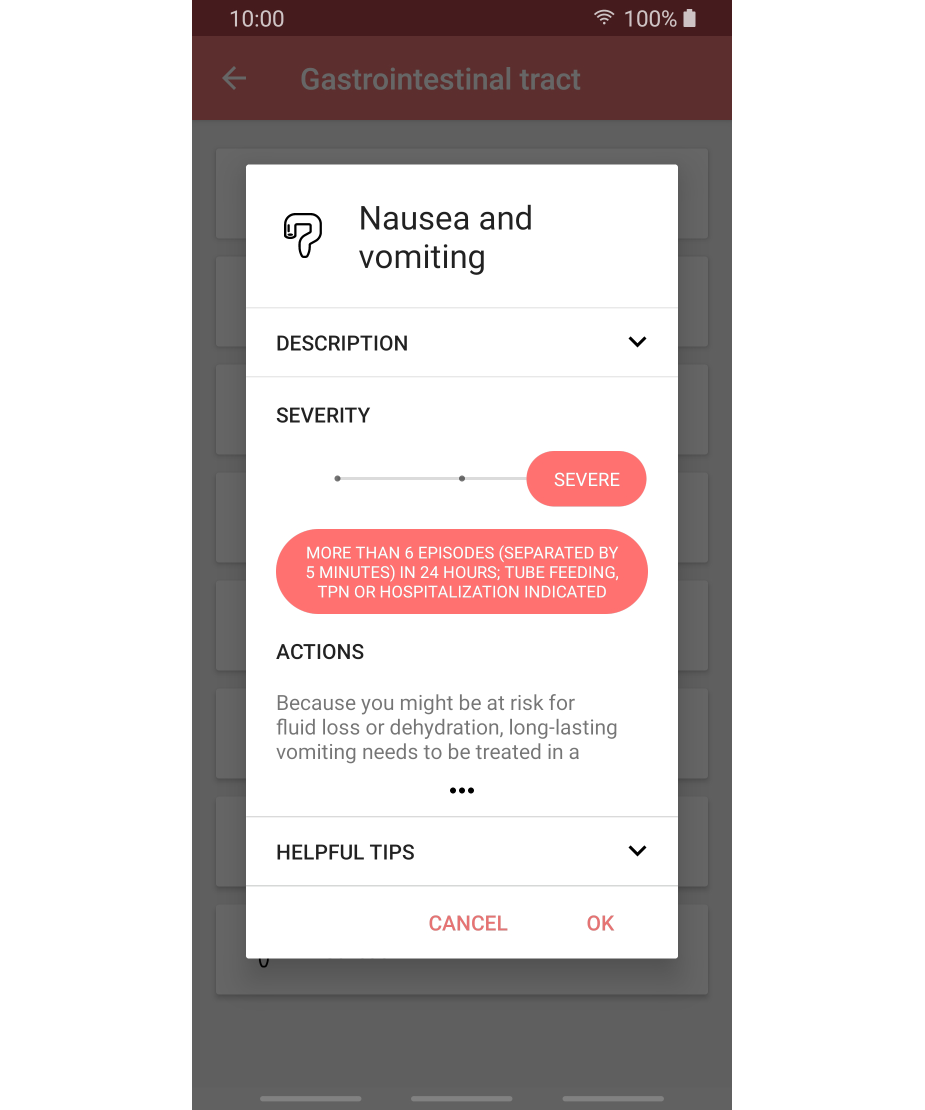

Supplement: Multimedia Appendix 2 [file mhealth_v8i8e17408_app2.png]

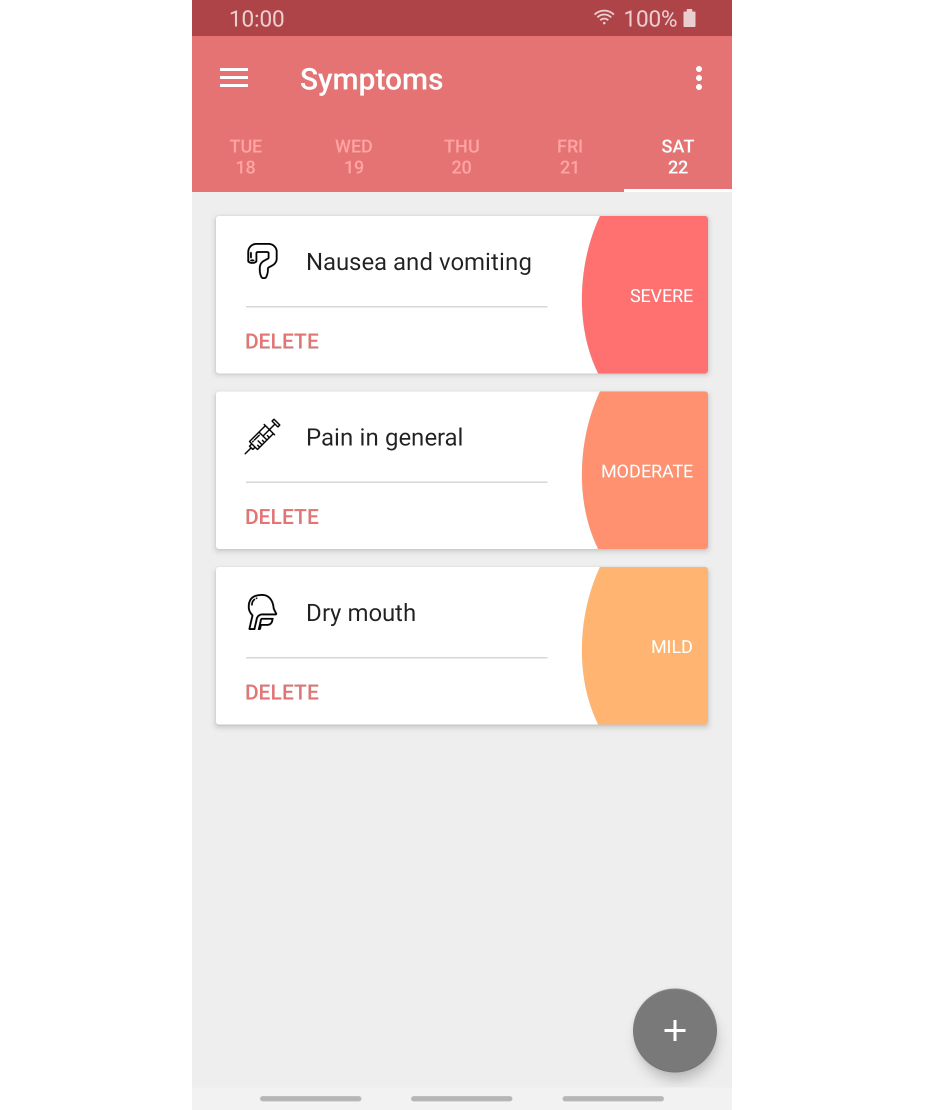

Supplement: Multimedia Appendix 3 [file mhealth_v8i8e17408_app3.png]
