# Supplementary material for: Mobile App for Symptom Management and Associated Quality of Life During Systemic Treatment in Early Stage Breast Cancer: Nonrandomized Controlled Prospective Cohort Study
Source: JMIR Mhealth Uhealth. 2020 Aug 4;8(8):e17408. doi: 10.2196/17408 (PMC7435681; doi:10.2196/17408)
Supplement: Multimedia Appendix 5 [file mhealth_v8i8e17408_app5.docx]

| **Variable** | **Estimate** | **95% CI** | ***P-value*** |
| --- | --- | --- | --- |
| (Intercept) | 10.1 | [-5.4, 25.6] | .20 |
| Time: First week | 10.6 | [4.8, 16.4] | <.001 |
| Time: End of treatment | 7.6 | [1.4, 13.8] | .02 |
| Group: mobile | 10.1 | [1.8, 18.5] | .02 |
| Mobile group × Time first week | -5.4 | [-13.6, 2.7] | .19 |
| Mobile group × Time end of treatment | -3.1 | [-12.6, 6.3] | .52 |
| Type of surgery: mastectomy | -1.1 | [-8.3, 6.1] | .76 |
| Quality of life at baseline | 0.6 | [0.4, 0.7] | <.001 |
