# Supplementary material for: Mobile App for Symptom Management and Associated Quality of Life During Systemic Treatment in Early Stage Breast Cancer: Nonrandomized Controlled Prospective Cohort Study
Source: JMIR Mhealth Uhealth. 2020 Aug 4;8(8):e17408. doi: 10.2196/17408 (PMC7435681; doi:10.2196/17408)
Supplement: Multimedia Appendix 6 [file mhealth_v8i8e17408_app6.docx]

| **Variable** | **Estimate** | **95% CI** | ***P-value*** |
| --- | --- | --- | --- |
| (Intercept) | 2.2 | [-20.9, 25.2] | .85 |
| Time: first week | 7.1 | [3.2, 11.0] | <.001 |
| Time: End of treatment | 2.3 | [-1.9, 6.4] | .28 |
| Group: intervention | 8.9 | [3.1, 14.7] | .003 |
| Intervention group × Time first week | -3.5 | [-9.0, 2.0] | .21 |
| Intervention group × Time end of treatment | 1.8 | [-4.6, 8.1] | .58 |
| Type of surgery: mastectomy | 1.6 | [-3.5, 6.8] | .53 |
| Summary score at baseline | 0.8 | [0.5, 1.0] | <.001 |
