# Supplementary material for: Mobile App for Symptom Management and Associated Quality of Life During Systemic Treatment in Early Stage Breast Cancer: Nonrandomized Controlled Prospective Cohort Study
Source: JMIR Mhealth Uhealth. 2020 Aug 4;8(8):e17408. doi: 10.2196/17408 (PMC7435681; doi:10.2196/17408)
Supplement: Multimedia Appendix 7 [file mhealth_v8i8e17408_app7.docx]

|  | **Before chemotherapy** | | **Change in the first week** | | | | **Change in the first cycle** | | | | **Change at the end of chemotherapy** | | | |
| --- | --- | --- | --- | --- | --- | --- | --- | --- | --- | --- | --- | --- | --- | --- |
|  | **I (n=46)** | **C (n=45)** | **I (n=46)** | **C (n=45)** | *P* | *adj. P* | **I (n=44)** | **C (n=43)** | *P* | *adj. p* | **I (n=24)** | **C (n=36)** | *P* | *adj. p* |
| **Physical functioning** | 100 (93.3, 100) | 100 (86.7, 100) | -3.3 (-13.3, 0) | -13.3 (-26.7, 0) | .051 | 1 | -6.7 (-13.3, 0) | -6.7 (-16.7, 0) | .45 | 1 | 0 (-8.3, 0) | -20 (-20, 0) | .005 | .27 |
| **Role functioning** | 100 (83.3, 100) | 100 (66.7, 100) | -16.7 (-33.3, 0) | -16.7 (-50, -16.7) | .15 | 1 | 0 (-33.3, 0) | -16.7 (-33.3, 0) | .40 | 1 | 0 (-16.7, 0) | -25 (-54.2, -12.5) | .01 | .49 |
| **Emotional functioning** | 83.3 (75, 97.9) | 75 (58.3, 83.3) | 0 (-8.3, 8.3) | 0 (-16.7, 8.3) | .28 | 1 | 0 (-8.3, 8.3) | 0 (-8.3, 16.7) | .97 | 1 | 0 (-10.4, 16.7) | -8.3 (-25, 8.3) | .132 | 1 |
| **Cognitive functioning** | 100 (83.3, 100) | 100 (83.3, 100) | 0 (-16.7, 0) | -16.7 (-33.3, 0) | .11 | 1 | 0 (-16.7, 0) | -16.7 (-33.3, 0) | .44 | 1 | 0 (-16.7, 0) | -16.7 (-33.3, 0) | .089 | 1 |
| **Social functioning** | 100 (70.8, 100) | 83.3 (66.7, 100) | 0 (-16.7, 0) | -16.7 (-50, 0) | .001 | .04 | 0 (-16.7, 0) | -16.7 (-33.3, 0) | .005 | 0.29 | 0 (-4.2, 16.7) | -33.3 (-33.3, 0) | .003 | .14 |
| **Fatigue** | 11.1 (0, 22.2) | 22.2 (0, 33.3) | 22.2 (2.8, 41.7) | 33.3 (22.2, 44.4) | .27 | 1 | 11.1 (0, 33.3) | 22.2 (0, 33.3) | .71 | 1 | 22.2 (11.1, 33.3) | 27.8 (0, 55.6) | .392 | 1 |
| **Nausea and vomiting** | 0 (0, 0) | 0 (0, 0) | 16.7 (0, 16.7) | 16.7 (0, 33.3) | .08 | 1 | 0 (0, 16.7) | 0 (0, 16.7) | .21 | 1 | 0 (0, 0) | 0 (0, 16.7) | .55 | 1 |
| **Pain** | 0 (0, 16.7) | 0 (0, 16.7) | 0 (0, 16.7) | 16.7 (0, 33.3) | .03 | 1 | 0 (-16.7, 16.7) | 0 (0, 16.7) | .31 | 1 | 0 (-4.2, 16.7) | 16.7 (0, 33.3) | .005 | .29 |
| **Dyspnoea** | 0 (0, 0) | 0 (0, 0) | 0 (0, 0) | 0 (0, 0) | .78 | 1 | 0 (0, 0) | 0 (0, 0) | .59 | 1 | 0 (0, 33.3) | 0 (0, 33.3) | .886 | 1 |
| **Insomnia** | 0 (0, 33.3) | 33.3 (0, 33.3) | 0 (0, 25) | 0 (0, 33.3) | .13 | 1 | 0 (0, 8.3) | 0 (0, 33.3) | 0.66 | 1 | 0 (0, 33.3) | 0 (0, 33.3) | .63 | 1 |
| **Appetite loss** | 0 (0, 0) | 0 (0, 0) | 0 (0, 33.3) | 33.3 (0, 33.3) | .04 | 1 | 0 (0, 0) | 0 (0, 33.3) | 0.77 | 1 | 0 (0, 8.3) | 0 (0, 33.3) | .75 | 1 |
| **Constipation** | 0 (0, 0) | 0 (0, 33.3) | 0 (0, 33.3) | 33.3 (0, 33.3) | .51 | 1 | 0 (0, 0) | 0 (0, 33.3) | 0.25 | 1 | 0 (0, 0) | 0 (0, 0) | .99 | 1 |
| **Diarrhoea** | 0 (0, 0) | 0 (0, 0) | 0 (0, 0) | 0 (0, 0) | .87 | 1 | 0 (0, 0) | 0 (0, 0) | 0.99 | 1 | 0 (0, 0) | 0 (0, 0) | .50 | 1 |
| **Body image** | 100 (91.7, 100) | 95.8 (66.7, 100) | 0 (0, 0) | -4.2 (-16.7, 0) | .01 | 0.44 | 0 (-16.7, 0) | -8.3 (-22.9, 0) | 0.20 | 1 | -8.3 (-18.8, 0) | -16.7 (-33.3, 0) | .56 | 1 |
| **Sexual functioning** | 33.3 (0, 33.3) | 25 (12.5, 33.3) | 0 (0, 0) | 0 (-16.7, 0) | .30 | 1 | 0 (-16.7, 0) | 0 (-16.7, 0) | 0.56 | 1 | 0 (-16.7, 4.2) | 0 (-16.7, 0) | .34 | 1 |
| **Sexual enjoyment** † | 66.7 (58.3, 100), n=28 | 33.3 (33.3, 66.7), n=24 | 0 (0, 0), n=23 | 0 (0, 0), n=21 | / | / | 0 (0, 0), n=21 | 0 (-33.3, 0), n=17 | / | / | 0 (-33.3, 0), n=13 | 0 (-8.3, 0), n=12 | / | / |
| **Future perspective** | 66.7 (33.3, 66.7) | 33.3 (25, 66.7) | 0 (0, 25) | 0 (0, 33.3) | .86 | 1 | 0 (0, 33.3) | 0 (-33.3, 25) | 0.12 | 1 | 0 (-33.3, 33.3) | 0 (0, 33.3) | .76 | 1 |
| **Systemic therapy side effects** | 4.8 (0, 9.5) | 9.5 (4.8, 14.3) | 14.3 (6, 23.8) | 19 (9.5, 38.1) | .08 | 1 | 14.9 (4.8, 28.6) | 28.6 (15.5, 38.1) | 0.006 | .33 | 19 (9.5, 29.8) | 28.6 (14.3, 38.1) | .23 | 1 |
| **Breast symptoms** | 12.5 (8.3, 25) | 16.7 (8.3, 27.1) | -8.3 (-14.6, 0) | -8.3 (-16.7, 0) | .70 | 1 | -8.3 (-16.7, 8.3) | -8.3 (-16.7, 0) | 0.33 | 1 | -4.2 (-10.4, 8.3) | -8.3 (-16.7, 4.2) | .23 | 1 |
| **Arm symptoms** | 0 (0, 11.1) | 11.1 (11.1, 25) | 0 (-11.1, 0) | 0 (-11.1, 0) | .48 | 1 | 0 (-11.1, 0) | 0 (-11.1, 4.2) | 0.75 | 1 | 0 (-11.1, 2.8) | 0 (-11.1, 16.7) | .91 | 1 |
| **Upset by hair loss** † | 33.3 (16.7, 50), n=3 | 50 (25, 75), n=4 | 0 (0, 0), n=1 | 0 (0, 0), n=2 | / | / | 0 (-16.7, 16.7), n=3 | 16.7 (-8.3, 33.3), n=4 | / | / | NA (NA, NA), n=0 | -33.3 (-33.3, -33.3), n=1 | / | / |
